# RESPIRIAMO – Italian hospital surveillance for lower respiratory tract infections: a multicenter retrospective cohort study of respiratory syncytial virus-associated hospitalizations in children under 2 years of age

**DOI:** 10.1186/s12887-026-07177-8

**Published:** 2026-06-24

**Authors:** Elena Bozzola, Emanuela Piccotti, Enza D’Auria, Sandra Trapani, Anna Chiara Vittucci, Francesca Tirelli, Susanna Esposito, Diego Peroni, Antonietta Giannattasio, Giovanni Corsello, Raffaella Nenna, Raffaele Badolato, Alessandra Coscia, Anna Benedetta Del Signore, Alessandra Simonetti, Respiriamo Study Group, Livia Antilici, Federica Attaianese, Paolo Rossi, Marta Ferretti, Daniele Franzone, Michele Ghezzi, Laura Folgori, Vincenzo Guaia, Cecilia Basile, Alessandra Carmignani, Flaminia Ruberti, Roberto Grandinetti, Roberta Forestiero, Laura Dotta, Roberta Ragucci, Emiliano Barbieri, Marco Maglione, Eugenio Baraldi, Silvia Bressan, Domenico Cipolla, Chiara Martorana, Claudia Colomba, Maria Valentina Catania, Claudio Costantino, Gregorio Serra, Francesca Pulcinelli, Fabio Midulla, Laura Petrarca

**Affiliations:** 1 Pediatric Unit, Bambino Gesù Children’s Hospital, IRCCS, Piazza Di Sant’Onofrio, 4, Rome, 00165 Italy; 2 Pediatric Emergency Medicine, IRCCS, Istituto Giannina Gaslini, Genoa, Italy; 3 Department of Pediatrics, Buzzi Children’s Hospital, University of Milan, Milan, Italy; 4 Department of Biomedical and Clinical Sciences, University of Milan, Milan, Italy; 5 Department of Health Sciences, University of Florence, Florence, Italy; 6 Meyer Children’s Hospital IRCCS, Paediatric Unit, Florence, Italy; 7 Department of Women’s and Children’s Health, Hospital-University of Padua, Padua, Italy; 8 Pediatric Clinic, University Hospital of Parma, Parma, Italy; 9 Azienda Ospedaliera, UO Pediatric Clinic, Universitaria Di Pisa”, Pisa, Italy; 10 Pediatric Emergency Department, Santobono-Pausilipon Children’s Hospital, Naples, Italy; 11 A.R.N.A.S. Ospedali Civico Di Cristina Benfratelli, Palermo, Italy; 12 Department of Health Promotion, Maternal and Child Health, Internal Medicine, and Specialized Medicine of Excellence “G. D’Alessandro”, University of Palermo, Palermo, Italy; 13 Department of Maternal Child and Urological Sciences, Sapienza University of Rome, Rome, Italy; 14 Department of Pediatrics of the “Spedali Civili Di Brescia”, Brescia, Italy; 15 Department of Public Health and Pediatric Sciences, University of Turin, Turin, Italy; 16 INCiPiT, Italian Network for Paediatric Clinical Trials, Rome, Italy; 17 UOC Trials, IRCCS “Bambino Gesù” Children’s Hospital, Rome, Italy; 18 Department of Systems Medicine, University of Rome Tor Vergata, Rome, Italy

**Keywords:** Infant, Respiratory syncytial virus, Hospitalization, Low respiratory trait infection, Oxygen, Intensive care unit, Monoclonal antibodies

## Abstract

**Background:**

Lower respiratory tract infections (LRTIs) are a leading cause of hospitalization in infants, with respiratory syncytial virus (RSV) representing the most important etiologic agent in children under 2 years of age. Although RSV disease burden is well recognized, multicenter data describing hospitalization characteristics, severity, and risk profiles in Italian infants remain limited, particularly following changes in viral circulation patterns in recent years. The study was established to provide robust hospital-based surveillance data. This study aimed to describe the clinical features and the severity of RSV-associated LRTI hospitalizations in Italian children under 24 months, comparing RSV-positive and RSV-negative cases.

**Methods:**

The study included infants aged 0– < 24 months hospitalized for LRTI between the 2018/2019 and 2020/2021 seasons across 12 Italian pediatric centers. Demographic characteristics, medical history, clinical presentation, laboratory radiologic, and hospitalization data were collected. Patients underwent RSV testing on nasopharyngeal samples and were classified as RSV-positive or RSV-negative. Descriptive statistics and comparative analyses were performed using parametric or non-parametric tests.

**Results:**

One thousand five hundred forty-seven infants were included, out of which 1,263 (81.6%) were RSV-positive. RSV-positive infants were significantly younger than RSV-negative infants, more likely to be previously healthy and without a family history of atopy. RSV infection was associated with a more severe clinical course, higher rates of oxygen supplementation (67.6% vs. 39.1%), respiratory support, invasive ventilation, and intensive care unit admission (10.6% vs. 3.1%). Use of RSV prophylaxis (at that time, palivizumab) was rare in both groups.

**Conclusions:**

RSV was the predominant cause of LRTI and was associated with significantly greater disease severity. Most hospitalized infants were previously healthy, underscoring the need for preventive strategies targeting the general infant population.

**Supplementary Information:**

The online version contains supplementary material available at https://doi.org/10.1186/s12887-026-07177-8.

## Background

Lower respiratory tract infections (LRTIs) represent one of the leading causes of morbidity and hospital admission in infants and young children worldwide. Among these, respiratory syncytial virus (RSV) is the single most significant pathogen responsible for bronchiolitis and viral pneumonia, particularly in children under the age of two. RSV is estimated to cause 33 million episodes of LRTIs and more than 3 million hospitalizations each year among children under 5 years, across the world [1]. While disease mortality is highest in low- and middle-income countries, medical assistance, like hospitalization, is highest in high-income settings, including Europe.

In recent years, Italy has contributed several epidemiological studies showing high RSV activity, significant health-care utilization, and frequent hospitalization in previously healthy infants. A recent nationwide analysis estimated that RSV accounts for 175–195 hospitalizations per 100,000 children aged 0–5, with the highest rates among infants aged < 1 year [2]. Notably, approximately 85% of RSV-hospitalized infants were previously healthy and full-term, highlighting that RSV is not limited to classical high-risk groups of infants [2].

However, significant gaps remain regarding fine-grained epidemiology in Italian infants < 2 years, including hospital-level burden and severity indicators. Although regional variability was not a pre-specified objective, the present study provides a center-level distribution of RSV-positive and RSV-negative cases across participating sites, offering insight into geographic variability. (Table A in supplementary material) Moreover, changes in viral circulation following the COVID-19 pandemic altered RSV seasonal dynamics in many countries, emphasizing the need for a robust, multicenter surveillance system [3, 4].

To address these gaps, the INCiPiT Consortium (Italian Network for Paediatric Clinical Trials) established RESPIRIAMO, an Italian hospital surveillance project integrating retrospective and prospective data on LRTI hospitalizations and RSV detection in infants < 24 months. The retrospective component covered three consecutive seasons (2018/2019, 2019/2020, 2020/2021) across 12 major Italian pediatric centers.

This article provides a scientific analysis of the retrospective dataset, encompassing demographics, clinical features, medical history, laboratory testing, and complications during hospitalization, comparing RSV-positive patients with those who are RSV-negative.

The results contribute to the understanding of RSV burden in Italy and support the development of preventive strategies, including monoclonal antibody prophylaxis and maternal vaccination—both recently recommended by global health authorities [5, 6].

## Materials and methods

### Study design

#### Study population and setting

Data were collected across major Italian pediatric hospitals and departments located in Rome (Bambino Gesù; Umberto I), Milan, Parma, Pisa, Palermo, Naples, Genoa, Padua, Brescia, Florence, and Turin. These sites were distributed across the entire national territory. Children were enrolled in the study if the following inclusion criteria were satisfied:

1. signed and dated written informed consent obtained from the parent(s)/legal representative(s) of the subject,
2. infants of 0- < 2 years,
3. hospitalization for LRTI during the 2018–2021 seasons; patients were included during three consecutive RSV seasons (2018–2019, 2019–2020, and 2020–2021), primarily covering the epidemic months (October to March). A limited number of cases were also recorded outside the epidemic period (April to September), (Table B in Supplementary material).
4. RSV testing via nasopharyngeal aspiration (NPA) or nasal/throat swab.

All sites followed local protocols for testing and clinical management.

#### Virological diagnosis

Nasopharyngeal aspirates or nasal/throat swabs were collected at hospital admission in accordance with local clinical protocols at all participating sites. Sample collection, transport, and storage followed each institution’s internal standards for respiratory virus detection; laboratory procedure was not standardizable, given the retrospective, multicenter design of the study. This variability is acknowledged as a methodological limit in the “Strengths and Limitations” section.

RSV detection was performed using either rapid antigen tests or nucleic acid amplification (PCR) methods, depending on the sites. Rapid diagnostic tests included [e.g., Sofia RSV FIA, Abbott BinaxNOW RSV, etc.], all authorized for clinical use in Italy, with reported sensitivities of approximately 80–90% and specificities > 95%.

In the following sites, in Rome (Bambino Gesù; Umberto I), Milan, Parma, Pisa, Palermo, Naples, Genoa, Padua, Brescia, Florence and Turin, a commercial multiplex real-time PCR assay (e.g., Allplex™ Respiratory Panel 4, Seegene; BioFire® FilmArray Respiratory Panel), detecting RSV A and B and a panel of other respiratory pathogens (influenza A/B, parainfluenza viruses, human metapneumovirus, adenovirus, rhinovirus/enterovirus, seasonal coronaviruses) has been employed. Finally, the panel of viruses tested was the same for all sites. Details about virological diagnosis have been summarized in Supplementary Table C.

#### Data sources

Data were anonymously collected through questionnaires completed by clinicians and parents to create the study dataset. The dataset included: demographic data (age and sex), diagnostic testing (rapid antigen tests, multiplex Polymerase Chain reaction-PCR), clinical parameters at presentation, medical history (prematurity defined as below 37 weeks, comorbidities, allergies, and family history), hospital course (oxygen supplementation, Intensive care unit (ICU) transfer, ventilation), laboratory findings and radiology. Patients were classified as RSV-positive or RSV-negative according to the results of RSV testing.

#### Statistical analysis

The primary outcome of disease severity was defined as the need for intensive care unit (ICU) admission and/or mechanical ventilation. Secondary severity outcomes included the need for supplemental oxygen, duration of oxygen therapy, use of high-flow oxygen therapy (HFNC) and non-invasive ventilation (NIV), as well as the occurrence of complications during hospitalization. Descriptive statistics were used: Means ± SD, medians with IQR for quantitative variables and proportions for categorical variables. Comparisons between RSV-positive and RSV-negative groups relied on the chi-square test for categorical variables, and Student's t-test or Mann–Whitney U test for continuous variables; *p*-values derived from your provided spreadsheet (*p*-value results).

Descriptive statistics were used: means ± standard deviation (SD) or medians with interquartile range (IQR) for continuous variables, and frequencies and percentages for categorical variables. Comparisons between RSV-positive and RSV-negative groups were performed using the chi-square test for categorical variables and Student’s t-test or Mann–Whitney U test for continuous variables, as appropriate based on data distribution. All p-values were derived from the statistical tests described above. Statistical analyses were performed using SAS software (version 9.4).

## Results

A total of 1547 infants with LRTI and RSV testing were included, of which 1263 (81.6%) were RSV-positive and 284 (18.4%) RSV-negative. Demographic data and clinical characteristics at presentation are summarised in Table 1. Further results are presented in tables included as supplementary material (Table D in supplementary materials).

**Table 1:** Demographic data and clinical characteristics of the cohort

Parameter	RSV-positive	RSV-negative	*p*-value*
Age, months, median (IQR)	2.0 (1–4)	3.0 (1.5–8)	< 0.0001
Sex (male/female), n (%)	682 (54%)/581 (46%)	168(59.1%)/116 (40.9%)	0.0082
Gestational age, weeks, mean ± SD	34.4 ± 3.7	33.6 ± 3.5	0.2186
Prematurity, n (%)	139 (11.1%)	37 (13.2%)	0.3332
Any Breastfeeding, n (%)	703 (56.3%)	152 (54.1%)	0.0106
Coinfections:			
Influenza, n (%)	11 (0.9%)	9 (3.2%)	0.2457
Sars-CoV2, n (%)	9 (0.7%)	4 (1.4%)	0.0019
Weight, Kg, mean ± SD	5.34 ± 1.8	5.50 ± 2.3	0.5252
Oxygen saturation at presentation, %, median (IQR)	97 (94–99)	97 (94–99)	0.1156
Respiratory rate at presentation, breaths/min mean ± SD	50.3 ± 12.8	52.0 ± 11.0	0.0475
Temperature at admission °C, mean ± SD	37 ± 0,8	37.1 ± 1,0	0.383

Continuous variables are presented as mean ± SD; categorical variables as N (%) and interquartile range

^*^P-values were derived using Student’s t-test for normally distributed variables and the Mann–Whitney U test for non-normally distributed variables

Comorbidities and underlying conditions are summarized in Table 2. Overall, the prevalence of comorbidities was low in both groups, although some differences were observed. RSV-negative patients more frequently reported a family history of atopy/asthma (21.7% vs. 9.2%) and previous respiratory infections requiring therapy (17.4% vs. 6.4%). Conversely, the prevalence of other conditions, including recurrent wheezing, bronchial asthma, food allergy, malnutrition, and immunocompromised status, was low and comparable between groups.

**Table 2:** Comorbilities by cohort

Parameter	RSV Positive (*N* = 1263)	RSV Negative (*N* = 284)
Any comorbidity, n (%)	67 (5.4%)	20 (7.1%)
Family history of atopy/asthma/eczema, n (%)	115 (9.2%)	61 (21.7%)
Recurrent wheezing, n (%)	10 (0.8%)	4 (1.4%)
Bronchial asthma, n (%)	2 (0.2%)	0
Neurodermatitis, n (%)	0	0
Food allergy, n (%)	5 (0.4%)	0
Immunocompromised status, n (%)	0	1 (0.4%)
Malnutrition, n (%)	3 (0.2%)	0
Previous respiratory infections requiring therapy, n (%)	80 (6.4%)	49 (17.4%)
Previous hospitalization for respiratory infection, n (%)	40 (3.2%)	32 (11.4%)

A significant difference between the two groups was observed regarding a family history of atopy, eczema, or asthma, which was less frequent among RSV-positive compared with RSV-negative patients (RSV + vs RSV-: 9.2% vs 21.7%) (*p* < 0.001).

As for personal history, comorbidities and chronic conditions were uncommon in all the infants (67 in the RSV + and 20 in the RSV- group), supporting the observation that most hospitalized infants were previously healthy. In line, only a small proportion patients in either group had received Palivizumab prophylaxis in both groups (RSV + vs RSV-:0.4% vs1.8%).

Regarding hospital course, the RSV-positive children were significantly more likely to require supplemental oxygen (*p* < 0.001). Oxygen therapy was required by 67.6% of the RSV + sample size and by 39.1% of the RSV- one.

In line, RSV positivity was associated with a more than threefold increased risk of ICU admission and a longer length of stay. In fact, 10.6% of RSV + and 3.1% of RSV- groups required intensive care support (*p* < 0.01) for a median ICU stay duration of 5.9 ± 4.1 days in the RSV positive group and 4.2 ± 2.8 days in the RSV negative– group (*p* = 0.118).

Ventilation support was higher in patients who tested positive for RSV (RSV +/RSV-: 28.4%/12.3%), with an almost threefold higher invasive ventilation support (RSV +/RSV-: 22.7%/8.4%) and of high-flow oxygen (RSV +/RSV-: 16.1%/6.7%) (*p* < 0.001). RSV-positive patients also showed a higher need for supplemental oxygen (67.6% vs. 39.1%) and a longer duration of oxygen therapy (median 96 vs. 72 h). In addition, complications were more frequent among RSV-positive patients (3.1% vs. 0.4%). Data are presented as supplementary material (Tables E and F).

To further investigate factors associated with disease severity, univariate and multivariate logistic regression analyses were performed (Tables G and H supplementary material). In univariate analysis, RSV-positive status was significantly associated with an increased risk of severe outcome (OR 2.82, 95% CI 1.95–4.10, *p* < 0.001), while no statistically significant associations were observed for age, sex, prematurity, or comorbidity.

In multivariate analysis, RSV-positive status remained independently associated with a higher risk of severe outcome (adjusted OR 2.67, 95% CI 1.73–3.70, *p* < 0.001). Other variables were not significantly associated, although prematurity showed a non-significant trend toward increased risk (adjusted OR 1.37, 95% CI 0.96–1.97, *p* = 0.084).

RSV-positive patients showed a higher proportion of severe outcomes compared to RSV-negative patients both overall (29.2% vs 12.7%, *p* < 0.001) and after stratification by comorbidity status (without comorbidities: 29.0% vs 12.7%, *p* < 0.001; with comorbidities: 35.0% vs 13.3%, *p* = 0.021), indicating that the increased severity associated with RSV infection is independent of underlying medical conditions (Tables I and Fig. 1 in Supplementary material).

Laboratory test data were available only as aggregated information on test utilization, without quantitative values, and were broadly comparable between groups. Complications were collected as categorical variables. RSV-positive patients showed higher rates of oxygen supplementation (67.6% vs 39.1%), longer duration of oxygen therapy (median 96 vs 72 h), ICU admission (10.6% vs 3.2%), longer ICU stay (median 5 vs 3 days), and post-ICU hospitalization (7.1% vs 1.4%). Overall complications were also more frequent among RSV-positive patients (3.1% vs 0.4%). Data are presented as supplementary material (Table L in supplementary materials).

## Discussion

RSV remains one of the most important pathogens responsible for severe LRTIs in infants worldwide. The RESPIRIAMO multicenter Italian cohort presented here provides a nationwide hospital-based dataset of RSV-associated LRTIs in children under 24 months within an Italian healthcare setting across three consecutive seasons (2018–2021). Several relevant findings emerge from this large retrospective analysis, each of which contributes to the growing understanding of RSV severity patterns and health-care burden. When interpreted in the context of new preventive strategies—including maternal vaccination and long-acting monoclonal antibodies- the results carry substantial implications for clinical practice, public health planning, and pediatric infectious disease policy in Italy and beyond.

Most RSV-hospitalized infants were previously healthy, as well as in literature [7, 8]. Prematurity, immunodeficiency, congenital heart disease, or chronic lung disease were rare, representing < 10%. This supports evidence that RSV causes severe disease even in healthy, full-term infants. A notable finding was the higher prevalence of family history of atopy, eczema, or asthma in the RSV-negative group. Epidemiological evidence indicates that RSV LRTIs tend to occur more frequently among previously healthy, non-atopic infants, whereas atopic predisposition is more commonly associated with rhinovirus infections and recurrent wheezing phenotypes [9]. The RSV negative group had more atopic background, consistent with RSV infection being less associated with atopy than other viral wheezing illnesses [10]. Our observation that RSV-negative infants had more recurrent wheezing and an atopic background further supports this distinction, in line with the observation that RSV often causes severe disease in non-wheezing infants with no underlying airway hyper-responsiveness [11, 12].

These findings help contextualize the natural history of respiratory morbidity: RSV bronchiolitis typically represents an acute, severe first episode, while rhinovirus bronchiolitis often indicates an underlying predisposition toward obstructive airway disease. Our data are consistent with this paradigm and in line with international pediatric cohorts.

Furthermore, our data show that RSV-positive infants were significantly younger than RSV negative infants. Age is a critical determinant of RSV severity: younger infants have smaller airways, reduced collateral ventilation, and more compliant thoracic structures, predisposing them to more severe obstruction when infected. All infants during their first RSV season are at risk and need protection, as recommended by international scientific societies and as implemented by most countries with nirsevimab [13–15]. Furthermore, several recent multicenter analyses have confirmed that infants younger than three months are even at higher risk for respiratory failure, ICU admission, and the need for mechanical ventilation [16, 17]. The RESPIRIAMO cohort confirms this trend with RSV-positive infants exhibiting higher rates of oxygen therapy requirement (68% vs. 39%), greater overall need for respiratory support (28% vs. 12%), a nearly threefold greater need for invasive ventilation and more frequent ICU admission (10.6% vs. 3.1%). These findings are consistent with recent European ICU studies showing that RSV is the most common identified virus among infants admitted with acute respiratory failure [18]. Importantly, despite advances in high-flow nasal cannula therapy and non-invasive respiratory support, the demand for intensive care remained substantial in our cohort.

Low palivizumab utilization was in line with Italian guidelines. Prophylaxis rates were minimal (< 2%), emphasizing that only a small proportion of high-risk infants were protected [19, 20]. In our sample, palivizumab use was rare (< 2%), consistent with Italian and European recommendations before the approval of nirsevimab. This supports the discussion of expanded RSV prevention to all infants entering their first RSV season and to those infants remaining vulnerable to RSV aged ≤ 24 months at the start of the season. Recent trials and observational data demonstrated that nirsevimab reduces RSV-related medical care and hospitalization by approximately 80% [21, 22]. As of January 2026, real-world evidence studies are available, including a systematic literature review and meta-analysis with data from 5 countries (France, Italy, Luxembourg, Spain, and the USA) that demonstrated similar to higher effectiveness in preventing RSV medical assistance and hospitalization in all infants entering their first RSV season [23]. Furthermore, studies conducted in the USA demonstrated even higher effectiveness in preventing RSV medical assistance and hospitalization in all infants by 89% and 98%, respectively [24, 25]. Given the high burden observed in RESPIRIAMO, universal preventive strategies can reduce the burden of RSV disease in Italy, as already demonstrated by several studies [26–28]. Maternal RSV vaccination is a highly effective preventive strategy as well that leverages passive immunity to protect newborns during their most vulnerable period, and it’s becoming a cornerstone of RSV prevention alongside infant monoclonal antibody programs.

Moreover, the consistent association between RSV positivity and a more severe clinical course has important implications, including the need for an early identification of infants at higher risk of adverse outcomes [29]. Younger infants with RSV should be carefully monitored for early markers of respiratory failure, such as increasing work of breathing, feeding difficulties, and hypoxemia. The tripling of ICU admission risk in RSV-positive infants highlights the need for adequate PICU capacity, especially during peak seasons [30]. International studies estimate RSV accounts for up to 50% of winter pediatric ICU occupancy [31]. This study is in line with existing literature, underlying the role of prevention. Our findings are consistent with previous international studies showing that RSV-positive respiratory infections are associated with younger age, greater lower respiratory tract involvement, and increased healthcare utilization compared with RSV-negative cases. While similar patterns have been reported in Northern Europe and other high-income settings, our study adds region-specific evidence from Southern Europe. This contributes to a more comprehensive understanding of RSV burden and may inform the implementation of emerging preventive strategies in Italy. Finally, the study reveals a public health implication of universal RSV prevention. The RESPIRIAMO data strongly support the need for broad preventive strategies.

### Strengths and limitations of the present study

Strengths of the study include a large sample size (over 1,500 infants across 12 centers), geographically diverse representation of Italian pediatric hospitals, and clinical and respiratory support data.

Limitations of the study include retrospective design, variation in testing methods (use of both antigen tests and PCR may have influenced RSV detection sensitivity), lack of viral load quantification or genotype assessment, further microbiological test rather than those described, and other variables (viral, maternal, environmental, host-related, nutritional, genetic) could influence disease progression. Additionally, we did not examine the epidemiological data.

Despite these limitations, the RESPIRIAMO dataset provides robust, clinically meaningful insights into RSV disease burden and severity in Italy.

## Conclusions

The RESPIRIAMO multicenter retrospective cohort provides compelling evidence that RSV is the major cause of LRTI hospitalizations in Italian infants under 2 years. Infants with RSV were younger, required more respiratory support, had higher ICU admission rates, and demonstrated a more severe clinical course than RSV-negative infants.

Most hospitalized infants were previously healthy, indicating that RSV prevention strategies should target all infants, not only traditional high-risk groups.

These data strongly support ongoing national and international efforts to expand RSV prevention programs, which have already demonstrated significant effectiveness and public health impact in terms of disease burden reduction at the population level.

## Supplementary Information

Supplementary Material 1.

## Data Availability

Data and material are available at dr Bozzola’s office.

## Abbreviations

LRTI: Low respiratory trait infection
RSV: Respiratory syncytial virus
ICU: Intensive care unit
