# Supplementary material for: RESPIRIAMO – Italian hospital surveillance for lower respiratory tract infections: a multicenter retrospective cohort study of respiratory syncytial virus-associated hospitalizations in children under 2 years of age
Source: BMC Pediatr. 2026 Jun 24;26:853. doi: 10.1186/s12887-026-07177-8 (PMC13560284; doi:10.1186/s12887-026-07177-8)
Supplement: Supplementary file 1 [file 12887_2026_7177_MOESM1_ESM.docx]

Table A. Regional distribution

Table B: Enrollment period

| **Year** | **Period** | **Statistics** | **RSV Positive (N=1263)** | **RSV Negative (N=284)** |
| --- | --- | --- | --- | --- |
| 2018 | Months: Jan–Mar | N (%) | 16 (1.3%) | 6 (2.1%) |
|  | Months: Oct–Dec | N (%) | 157 (12.4%) | 52 (18.3%) |
|  | Other months: Apr–Sep | N (%) | 2 (0.2%) | 6 (2.1%) |
| 2019 | Months: Jan–Mar | N (%) | 299 (23.7%) | 69 (24.3%) |
|  | Months: Oct–Dec | N (%) | 123 (9.7%) | 23 (8.1%) |
|  | Other months: Apr–Sep | N (%) | 11 (0.9%) | 6 (2.1%) |
| 2020 | Months: Jan–Mar | N (%) | 197 (15.6%) | 21 (7.4%) |
|  | Months: Oct–Dec | N (%) | 2 (0.2%) | 24 (8.5%) |
|  | Other months: Apr–Sep | N (%) | 2 (0.2%) | 1 (0.4%) |
| 2021 | Months: Jan–Mar | N (%) | 2 (0.2%) | 16 (5.6%) |
|  | Months: Oct–Dec | N (%) | 426 (33.7%) | 54 (19.0%) |
|  | Other months: Apr–Sep | N (%) | 14 (1.1%) | 5 (1.8%) |
| 2022–2023 | Months: Jan–Mar | N (%) | 12 (1.0%) | 1 (0.4%) |

Table C Virilogical Data

| **Site** | **Sample type** | **Collection protocol standardized (Y/N)** | **Transport & storage standardized (Y/N)** | **Rapid test consistent across sites (Y/N)** | **Reported sensitivity (%)** | **PCR performed (Y/N)** | **PCR strategy (all samples vs only negative rapid tests)** | **Type of PCR assay (commercial/in-house)** | **Multiplex PCR details (panel, manufacturer)** | **Respiratory viruses  included in multiplex panel** | **Other viruses tested** | **Same viral panel across sites (Y/N)** | **Samples tested in duplicate (Y/N)** |
| --- | --- | --- | --- | --- | --- | --- | --- | --- | --- | --- | --- | --- | --- |
| Roma Bambino Gesù | Nasopharyngeal swab | Y | Y | Y (Beta Diagnostics: rapid immunochromatographic test for RSV) | Sensitivity > 95% | Y | All samples | Commercial | *Allplex™ Respiratory Full Panel Assay | FluA, FluB, RSV, PIVs 1-4, HRV/EV, HMPV, SARS-CoV-2, HCoV, MERS, BORp, BORpa, Myc, Cla, | None | N | N |
| Milano | Nasopharyngeal swab | Y | Y | Y (Beta Diagnostics: rapid immunochromatographic test for RSV) | Sensitivity > 95% | Y | All samples | Commercial | FilmArray Respiratory Panel (FilmArray® Respiratory Panel, BioFire Diag-nostics LLC, 390 Wakara Way, Salt Lake City, UT, USA) | FluA, FluB, RSV, PIVs 1-4, HRV/EV, HMPV, SARS-CoV-2, HCoV, MERS, BORp, BORpa, Myc, Cla, | None | N | N |
| Parma | Nasopharyngeal swab | Y | Y | Y (Beta Diagnostics: rapid immunochromatographic test for RSV) | Sensitivity > 95% | Y | All samples | Commercial | FilmArray Respiratory Panel (FilmArray® Respiratory Panel, BioFire Diag-nostics LLC, 390 Wakara Way, Salt Lake City, UT, USA) | FluA, FluB, RSV, PIVs 1-4, HRV/EV, HMPV, SARS-CoV-2, HCoV, MERS, BORp, BORpa, Myc, Cla, | None | N | N |
| Pisa | Nasopharyngeal swab | Y | Y | Y (Beta Diagnostics: rapid immunochromatographic test for RSV) | Sensitivity > 95% | Y | All samples | Commercial | FilmArray Respiratory Panel (FilmArray® Respiratory Panel, BioFire Diag-nostics LLC, 390 Wakara Way, Salt Lake City, UT, USA) | FluA, FluB, RSV, PIVs 1-4, HRV/EV, HMPV, SARS-CoV-2, HCoV, MERS, BORp, BORpa, Myc, Cla, | None | N | N |
| Palermo | Nasopharyngeal swab | Y | Y | Y (Beta Diagnostics: rapid immunochromatographic test for RSV) | Sensitivity > 95% | Y | All samples | Commercial | FilmArray Respiratory Panel (FilmArray® Respiratory Panel, BioFire Diag-nostics LLC, 390 Wakara Way, Salt Lake City, UT, USA) | FluA, FluB, RSV, PIVs 1-4, HRV/EV, HMPV, SARS-CoV-2, HCoV, MERS, BORp, BORpa, Myc, Cla, | None | N | N |
| Napoli | Nasopharyngeal swab | Y | Y | Y (Beta Diagnostics: rapid immunochromatographic test for RSV) | Sensitivity > 95% | Y | Negative test according to availability | Commercial | FilmArray Respiratory Panel (FilmArray® Respiratory Panel, BioFire Diag-nostics LLC, 390 Wakara Way, Salt Lake City, UT, USA) | FluA, FluB, RSV, PIVs 1-4, HRV/EV, HMPV, SARS-CoV-2, HCoV, MERS, BORp, BORpa, Myc, Cla, | None | N | N |
| Genova | Nasopharyngeal swab | Y | Y | Y (Beta Diagnostics: rapid immunochromatographic test for RSV) | Sensitivity > 95% | Y | All samples | Commercial | FilmArray Respiratory Panel (FilmArray® Respiratory Panel, BioFire Diag-nostics LLC, 390 Wakara Way, Salt Lake City, UT, USA) | FluA, FluB, RSV, PIVs 1-4, HRV/EV, HMPV, SARS-CoV-2, HCoV, MERS, BORp, BORpa, Myc, Cla, | None | N | N |
| Brescia | Nasopharyngeal swab | Y | Y | Y (Beta Diagnostics: rapid immunochromatographic test for RSV) | Sensitivity > 95% | Y | All samples | Commercial | FilmArray Respiratory Panel (FilmArray® Respiratory Panel, BioFire Diag-nostics LLC, 390 Wakara Way, Salt Lake City, UT, USA) | FluA, FluB, RSV, PIVs 1-4, HRV/EV, HMPV, SARS-CoV-2, HCoV, MERS, BORp, BORpa, Myc, Cla, | None | N | N |
| Firenze | Nasopharyngeal swab | Y | Y | N | Sensitivity > 95% | Y | All samples | Commercial | TaqPath™ 1‐Step RT‐qPCR Master Mix(Life Technologies, The Netherland | FluA, FluB, RSV, PIVs 1-4, HRV/EV, HMPV, SARS-CoV-2, HCoV, MERS, BORp, BORpa, Myc, Cla, | None | N | N |
| Torino | Nasopharyngeal swab | Y | Y | Y (Beta Diagnostics: rapid immunochromatographic test for RSV) | Sensitivity > 95% | Y | All samples | Commercial | CE-IVD marked | FluA, FluB, RSV, PIVs 1-4, HRV/EV, HMPV, SARS-CoV-2, HCoV, MERS, BORp, BORpa, Myc, Cla, | None | N | N |
| Roma Umberto I | Nasopharyngeal swab | Y | Y | Y (Beta Diagnostics: rapid immunochromatographic test for RSV) | Sensitivity > 95% | Y | All samples | Commercial | TaqPath™ 1‐Step RT‐qPCR Master Mix(Life Technologies, The Netherland | FluA, FluB, RSV, PIVs 1-4, HRV/EV, HMPV, SARS-CoV-2, HCoV, MERS, BORp, BORpa, Myc, Cla, | None | N | N |
| Padova | Nasopharyngeal swab | Y | Y | Y (Beta Diagnostics: rapid immunochromatographic test for RSV) | Sensitivity > 95% | Y | All samples | Commercial | TaqPath™ 1‐Step RT‐qPCR Master Mix(Life Technologies, The Netherland | FluA, FluB, RSV, PIVs 1-4, HRV/EV, HMPV, SARS-CoV-2, HCoV, MERS, BORp, BORpa, Myc, Cla, | None | N | N |

* Allplex™ Respiratory Full Panel Assay – Real-Time PCR for the diagnosis of respiratory infections through the detection of 26 bacterial and viral pathogens. A comprehensive test for the detection and identification of 26 respiratory pathogens, based on a one-step real-time RT-PCR. Clinically significant respiratory infections caused by various viral and bacterial pathogens commonly occur in symptomatic adult and pediatric patients. In order to guide strategies for effective treatment and control the spread of disease, clinicians need to know which pathogens are present in the early stages of infection, even in cases of multiple infections. However, traditional methods are slow, incomplete, and inaccurate. Patients are often discharged without specific treatment, or are prescribed empirical antibiotic therapy, without the identification of the pathogens involved. A multiplex real-time PCR test that simultaneously detects both viral and bacterial pathogens is indicated to identify the source of infection with accuracy, high sensitivity, and reliability to improve patient care and avoid errors or overuse. The Allplex™ Respiratory Full Panel Assay kit is a one-step RT-real-time PCR multiplex test that simultaneously detects and identifies 16 viruses, 7 bacteria, and 3 subtypes of influenza A - all agents of respiratory diseases - in clinical samples. The kit is highly automatable, from sample extraction to PCR reaction setup. When automated, the total analysis time is 4 hours and 30 minutes, with a throughput ranging from 20 samples per run (4 panels) to 94 samples per run (1 panel).

| Table D. Chest X-ray |  |  |
| --- | --- | --- |
|  |  |  |
| **Chest X-ray** | | |
|  |  |  |
| **Parameter** | **RSV Positive (N=1263)** | **RSV Negative (N=284)** |
| Chest X-ray, n (%) | 409 (32.4%) | 84 (29.6%) |
| Number of chest X-rays, median (IQR) | 1.0 (1.0–1.0) | 1.0 (1.0–1.0) |

Table E. Oxygen and ICU admission

| **Parameter** | **RSV Positive (N=1263)** | **RSV Negative (N=284)** |
| --- | --- | --- |
| Oxygen supplementation | 67.6% | 39.1% |
| Oxygen duration (median, hours) | 96 | 72 |
| ICU admission | 10.6% | 3.2% |
| ICU stay (median, days) | 5 | 3 |
| Post-ICU hospitalization | 7.1% | 1.4% |
| Overall complications | 3.1% | 0.4% |

| Table F. ICU admission and oxygen supplementation |  |  |
| --- | --- | --- |
|  |  |  |
| **Outcome** | **RSV Positive (N=1263)** | **RSV Negative (N=284)** |
| ICU admission, n (%) | 134 (10.6%) | 9 (3.2%) |
| Mechanical ventilation, n (%) | 359 (28.4%) | 35 (12.3%) |
| Supplemental oxygen, n (%) | 854 (67.6%) | 111 (39.1%) |
| Duration of oxygen therapy (hours), median (IQR) | 96 (48–144) | 72 (24–120) |
| High-flow oxygen therapy (HFNC), n (%) | 203 (16.1%) | 19 (6.7%) |
| Non-invasive ventilation (NIV), n (%) | 72 (5.7%) | 11 (3.9%) |
| Complications, n (%) | 39 (3.1%) | 1 (0.4%) |

Tables G and H Univariate and multivariate analysis

| **Univariate logistic regression** | | |  |
| --- | --- | --- | --- |
|  |  |  |  |
|  |  |  |  |
| **Variable** | **OR (95% CI)** | **p-value** |  |
| RSV-positive (vs negative) | 2.82 (1.95–4.10) | <0.001 |  |
| Age (per 1-month increase) | 0.97 (0.95–1.00) | 0.087 |  |
| Male sex | 1.02 (0.81–1.28) | 0.887 |  |
| Prematurity | 1.30 (0.93–1.84) | 0.127 |  |
| Comorbidity | 1.26 (0.87–1.82) | 0.218 |  |
|  |  |  |  |
|  |  |  |  |
|  |  |  |  |
| **Multivariate logistic regression** | | |  |
|  |  |  |  |
| **Variable** | **Adjusted OR (95% CI)** | **p-value** |  |
| RSV-positive (vs negative) | 2.67 (1.73–3.70) | <0.001 |  |
| Age (per 1-month increase) | 0.98 (0.95–1.01) | 0.271 |  |
| Male sex | 1.06 (0.84–1.35) | 0.616 |  |
| Prematurity | 1.37 (0.96–1.97) | 0.084 |  |
| Comorbidity | 1.33 (0.91–1.94) | 0.150 |  |
|  |  |  |  |

Table I Outcome according to RSV+/RSV-

Table L . Outcome in RSV+ and RSV- groups

Figure 1. Bar chart - ICU patients by age group
